# Expeditious Discovery of Small-Molecule Thermoresponsive Ionic Liquid Materials: A Review

**DOI:** 10.3390/molecules28196817

**Published:** 2023-09-27

**Authors:** Hsin-Yi Li, Yen-Ho Chu

**Affiliations:** Department of Chemistry and Biochemistry, National Chung Cheng University, Chiayi 62102, Taiwan; lanbarla0708@gmail.com

**Keywords:** smart material, thermoresponsive ionic liquid, thermoresponsive zwitterionic liquid, zwitterionic material, combinatorial chemistry, affinity extraction

## Abstract

Ionic liquids (ILs) are a class of low-melting molten salts (<100 °C) constituted entirely of ions, and their research has gained tremendous attention in line with their remarkably growing applications (>124,000 publications dated 30 August 2023 from the Web of Science^TM^). In this review, we first briefly discussed the recent developments and unique characteristics of ILs and zwitterionic liquids (ZILs). Compared to molecular solvents and other conventional organic compounds, (zwitter) ionic liquids carry negligible volatility and are potentially recyclable and reusable. For structures, both ILs and ZILs can be systematically tailor-designed and engineered and are synthetically fine-tunable. As such, ionic liquids, including chiral, supported, task-specific ILs, have been widely used as powerful ionic solvents as well as valuable additives and catalysts for many chemical reactions. Moreover, ILs have demonstrated their value for use as polymerase chain reaction (PCR) enhancers for DNA amplification, chemoselective artificial olfaction for targeted VOC analysis, and recognition-based affinity extraction. As the major focus of this review, we extensively discussed that small-molecule thermoresponsive ILs (TILs) and ZILs (zwitterionic TILs) are new types of smart materials and can be expeditiously discovered through the structure and phase separation (SPS) relationship study by the combinatorial approach. Using this SPS platform developed in our laboratory, we first depicted the rapid discovery of N,N-dialkylcycloammonium and 1,3,4-trialkyl-1,2,3-triazolium TILs that concomitantly exhibited LCST (lower critical solution temperature) phase transition in water and displayed biochemically attractive *T*_c_ values. Both smart IL materials were suited for applications to proteins and other biomolecules. Zwitterionic TILs are ZILs whose cations and anions are tethered together covalently and are thermoresponsive to temperature changes. These zwitterionic TIL materials can serve as excellent extraction solvents, through temperature change, for biomolecules such as proteins since they differ from the common TIL problems often associated with unwanted ion exchanges during extractions. These unique structural characteristics of zwitterionic TIL materials greatly reduce and may avoid the denaturation of proteins under physiological conditions. Lastly, we argued that both rational structural design and combinatorial library synthesis of small-molecule TIL materials should take into consideration the important issues of their cytotoxicity and biosafety to the ecosystem, potentially causing harm to the environment and directly endangering human health. Finally, we would concur that before precise prediction and quantitative simulation of TIL structures can be realized, combinatorial chemistry may be the most convenient and effective technology platform to discover TIL expeditiously. Through our rational TIL design and combinatorial library synthesis and screening, we have demonstrated its power to discover novel chemical structures of both TILs and zwitterionic TILs. Undoubtedly, we will continue developing new small-molecule TIL structures and studying their applications related to other thermoresponsive materials.

## 1. Introduction

**Figure 1:**
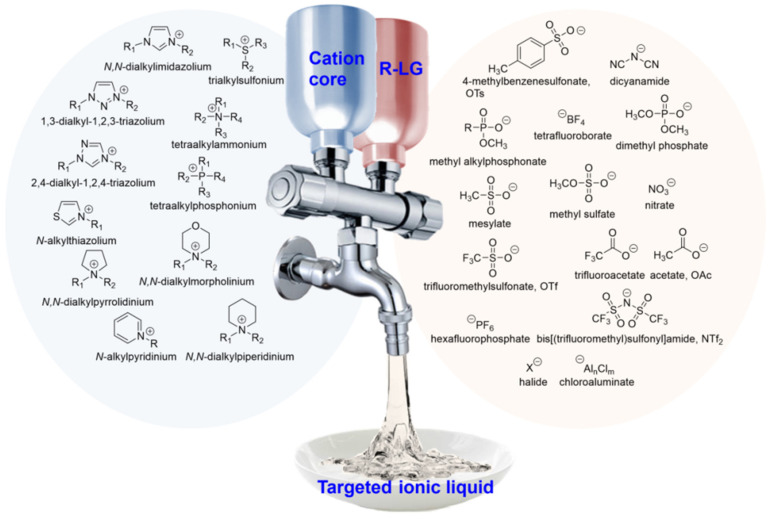
Ionic liquids are entirely composed of cations and anions.

Ionic liquids (ILs) are advanced materials entirely made of anion and cation pairs. They possess unique characteristics of diversity and controllability as ionic solvents, setting them apart from conventional molecular solvents known for their high volatility. Figure 1 shows the common anion and cation structures. ILs exhibit a plethora of outstanding properties, including extremely low vapor pressure, a low melting point, high polarity, scarce inflammability, and high thermal and chemical stability [1,2]. Additionally, as attractive electrolytes for batteries, ILs exhibit high conductivity, tunable electrochemical windows, and a wide liquid temperature range. Due to these remarkable properties and their recyclability, ILs have the potential to replace commonly used volatile organic solvents [3,4]. Because ILs conform to the principles of green chemistry and have great controllability in structural design, they have been widely studied and experimented with in various fields [5].

Throughout the development of ILs, we can roughly classify them into four stages. The first generation of ILs was extremely sensitive to moisture and air, and their structures were organic cations combined with metal halide anions, which has attracted attention due to their physical properties and electrochemical redox potential. The drawback was that these ILs easily decomposed or deteriorated when exposed to water or air [6]. The second generation of ILs is room-temperature ILs with high conductivity, which are stable in water and air [7], allowing scientists from various research fields to extensively explore imidazolium-based ILs. The third generation of ILs has abandoned the existing structural framework, and scientists have designed and synthesized functional ionic liquids and applied them in targeted fields [8,9]. The fourth and newest generation of ILs is being developed with the aim of applying them in biology, ecology, and medicine, and the ‘bio-safer’ IL structures are synthesized and investigated [10,11].

**Figure 2:**
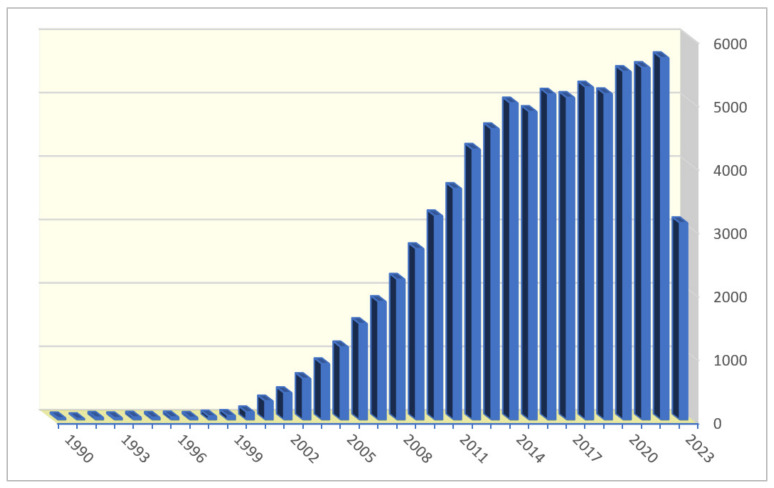
Number of papers published in English on the subject of ‘ionic liquids’, as determined by document type limit to ‘Journal’, ‘Review’, ‘Book’, ‘Letter’ using SciFinder^n^ dated up to 14 July 2023.

In the past twenty-two years, ILs have been developed vigorously, and the number of articles related to ILs has increased since 2000 and remained at greater than 5000 ever since 2016, as shown in Figure 2 (SciFinder electronic database and English journals within categories of book, journal, letter, and review).

## 2. Ionic Liquids Are Designable and Structurally Tunable

### 2.1. Ionic Liquids as Attractive Reaction Media

**Figure 3:**
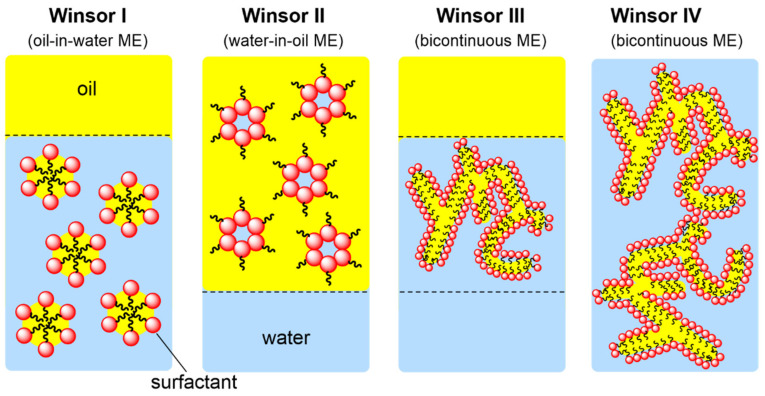
Winsor classification of microemulsions (ME).

With the aforementioned advantages, ILs can likely replace conventional molecular solvents. However, most ILs have the disadvantages of high viscosity and laborious purification. This drawback may be overcome by using ILs in microemulsion systems. Bica-Schröder and coworkers showed that microemulsion systems can be divided into four types (Figure 3) [12]: Winsor I and II are oil-in-water (o/w) and water-in-oil (w/o) microemulsion surfactant biphase systems, respectively. The mesophase of the Winsor III excess surfactant is between the water and oil phases, forming coexistence. Winsor IV is a single-phase isotropic solution formed by more surfactants. Compared to traditional emulsions, microemulsions not only have a significantly small structural size (3–30 nm) but also have thermodynamic stability, which can be converted into the long-term stability of mixed-polarity/non-polarity systems.

In addition, ILs possess the advantages of ultra-low vapor pressure, excellent solvation power, and high chemical and thermal stabilities, making them more suitable as solvents for chemical synthesis. For example, many natural and unnatural products carry quinazoline rings. Also, a good number of quinazoline derivatives are currently in different stages of clinical practice, and some have been sold as drugs. Based on bicyclic imidazolium- and triazolium-based ILs in combination with microwave-assisted organic synthesis, we successfully synthesized tryptanthrin, batracylin, and rutaecarpine using the rapid, one-pot, and high-yielding method and investigated their biological activities when appropriate [13,14,15].

### 2.2. Chiral Ionic Liquids and Supported Ionic Liquids

In the past decade, more and more attention has been paid to the development of chiral ionic liquids (CILs) and their applications in various separation technologies. The molecular diversity of CILs can be large as long as the anion or cation carries a chiral center or even has a few ionic liquids that have cationic and anion chiral pairs at the same time. This allows for the design of multi-variable structures. With the characteristics of chiral recognition, CILs play an important role in enantiomeric separation [16], for which the application scope of CILs has been expanded. In 2020, Flieger and coworkers published a review article on CILs [17] and introduced CILs as a new type of chiral solid material that was used for chiral recognition of enantiomeric analytes. The applications of CILs include various extraction techniques and their use as catalysts in chemical reactions, such as the Diels–Alder and aza-Baylis–Hillman reactions, which enhance the purity of enantiomeric products. In 2009, Bonanni published a simple and direct synthesis method to obtain novel chiral pyrrolidinium ILs starting with tartaric acid [18].

In addition, supported ILs can be used to enhance the reaction rate. Salunkhe carried out Knoevenagel condensation in various ILs and found that the Brønsted acidic IL, [Hmim][Tfa], enhanced the reaction rate [19]. Bazureau used IL-supported benzaldehyde and underwent condensation reactions with alkylamines, followed by a 1,3-dipolar cycloaddition reaction regioselectively. Finally, the IL could be readily removed using NaOMe to obtain the desired product, and the initial IL was released [20].

### 2.3. Zwitterionic Ionic Liquids

Ohno reported for the first time the synthesis of zwitterionic imidazolium sulfonate ILs (ZILs) in 2001 [21]. ZILs refer to ILs in which cationic and anion pairs are tethered together within the same structure. ZILs often exhibit high melting points. However, charge delocalization in ZILs helps reduce the melting point and viscosity. ZILs have been employed as electrochemical ionophores [21], reaction catalysts [22], and attractive solvents for dissolving cellulose [23]. In general, ZILs have opened a new chapter in developing specific chemical applications.

### 2.4. Task-Specific Ionic Liquids for CO_2_ Capture

Carbon dioxide has a significant impact on the environment and is known to contribute to the greenhouse effect. Carbon capture and storage (CCS), or carbon capture and sequestration (CCS), is a widely recognized method for capturing carbon dioxide. Here, the carbon dioxide produced by petrochemical combustion is transported from its place of origin through pipelines or ships and finally stored deep underground in geological formations. However, the cost of CCS remains high, and it is difficult to compare it with that of renewable energy [24,25]. Therefore, it is critical to develop cost-effective capture technologies for carbon dioxide. ILs have been specifically applied to carbon dioxide capture, and this type of structural design generally aims to place basic groups at the end of side chains and make the target gas react with solutes in the liquid phase, which is the main mode of sequestration. For example, Davis and coworkers reported the use of ILs to capture carbon dioxide in a more convenient and safer manner compared to capture with conventional molecular solvents or alkaline substances [26]. They reported that the capture conversion rate of [AminoC3-b-im][BF_4_] to carbon dioxide could reach 49.6%, and the mass of carbon dioxide was increased by 7.4% at atmospheric pressure, which is far superior to that of [h-mim][PF_6_] (0.01%) [26]. In addition, Shukla and Mikkola (2019) summarized the technology of capturing carbon dioxide with ILs and the capture mechanism in detail. They discussed the feasibility of commercially capturing carbon dioxide with ILs [27].

### 2.5. Ionic Liquids as PCR Enhancers

Since its introduction in 1988, the polymerase chain reaction (PCR) has played a vital role in amplifying DNA fragments using thermophilic polymerases [28]. Today, PCR is widely and frequently used for medical and biological applications, such as gene duplication, disease diagnosis, and forensic identification [29]. Despite the considerable diagnostic potential of PCR, amplifying the GC-rich DNA sequences by PCR still poses a significant challenge. It is known experimentally that GC-rich DNAs are difficult to amplify using the standard PCR amplification procedures, which can be attributed to the secondary structure in DNA [30,31]. Although it is straightforward to enzymatically replicate GC-rich DNA in living organisms, improved technologies or new methods must be developed and implemented to replicate them in vitro. Literature reports have demonstrated that slowdown PCR or enhancers such as DMSO or betaine overcome such obstacles [30,32]. Our laboratory previously revealed that ILs promoted PCR amplification of GC-rich DNAs by reducing their melting temperatures (Tm) [33].

### 2.6. Functionalized Ionic Liquids

**Figure 4:**
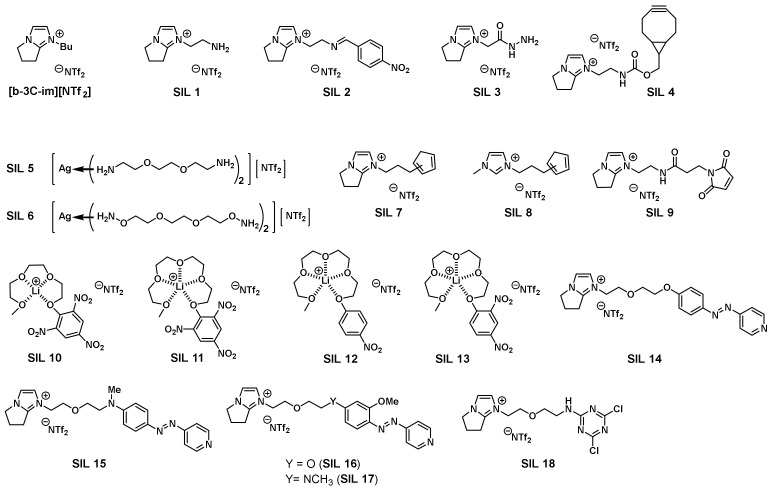
Structures for sensing ionic liquids were previously developed in our laboratory [40].

Chemoselective detection of volatile organic compounds (VOCs) in the environment has always been considered an important research topic. Human naked eyes and noses cannot identify directly and sense remotely the presence of minute amounts of natural or man-made gaseous, potentially hazardous substances. If the target VOCs in the environment can be experimentally measured and accurately determined, an early warning on the potential exposure to dangerous and unhealthy environments could then be timely disclosed [34]. With their nature of negligible vapor pressure and diversity in structural tunability, ILs have the potential to be tailor-synthesized and thin-coated on sensorchips in the mass-sensitive piezoelectric quartz crystal microbalance (QCM) to detect chemoselectively the target VOCs. A pioneering work on the relationship between the mass deposited and resonance frequency shift of quartz crystal was published by Sauerbrey [35], which was later widely used to measure changes in nanoscale masses. The first research on the use of ILs as sensing materials with QCM was published by Dai in 2002 [36]. Dai adopted quartz chips with gold electrodes on their surface and coated them with IL to detect volatile gases. The quartz chips can be regenerated by washing with acetonitrile. Since then, ILs have been used for gas detection on the QCM platform for more than two decades. Related applications include disease diagnosis (i.e., bacteria, viruses, and protein adsorption study) [37]; manufacturing industry (i.e., flammable and toxic gases monitoring); environmental protection (i.e., vehicle gas emissions and greenhouse gases measurement); indoor air quality monitoring; and homeland security (i.e., chemical and biological warfare agents). More than 20 VOCs detected on QCM have been published in reputable journals [38]. Sensing ILs detect and identify volatile molecules in real-time on the QCM platform, during which ionic liquids show supersensitivity performance [39]. In the last decade, we have also actively engaged in the development and establishment of a QCM-based chemoselective gas detection platform in our laboratory and continuously improved its detection capability for various gases. The advantage of our technology is that it uses a minute amount of tailor-prepared sensing ILs to thin-coat it on the chip, which reduces the risk of irreversible damage caused by direct chemical modification on the chip and reuses the chip to greatly reduce the detection cost. This technical platform can be realized because ILs are able to make gases more readily permeable than solids and have low vapor pressure to ensure that they will not dry up on the QCM chip during detection. In addition, the high polarity, chemical stability, and excellent solubility of ILs make our envisaged detection reactions proceed smoothly. At present, the developed sensing ILs have been applied to the detection of various target gases chemoselectively, which are summarized in Figure 4 [40].

### 2.7. Affinity Ionic Liquids

Ionic liquids can also be combined with microextraction technology using small amounts of samples and solvents to reduce the excessive waste produced during the process; one such example is applying ILs in affinity extraction based on biomolecular recognition. Biomolecular recognition can be defined as the specific binding between receptors and ligands. There are two reasons for its selectivity: geometrical configuration and noncovalent bonding attraction. The former is the appropriate mutual recognition orientation between the receptor and ligand in geometric size and shape; the latter is the recognition of interaction between two molecules through noncovalent bonding such as hydrogen bonds or π–π interactions. ILs are designer solvents, as they are not solvents with a single function or form but can be modified and tailor-functionalized according to the requirements of the experiments. Our laboratory reported an affinity ionic liquid (AIL) with molecular recognition function in 2009 [41] and developed a fast and highly efficient protein purification technology based on the affinity between biomolecules. This research uses fluorescent-tagged hexahistidine peptides FITC-(His)_6_-NH_2_ and His-tag green fluorescent protein (GFP) with AIL to establish separation and purification platforms, which can greatly improve the purification efficiency and shorten the purification duration.

**Figure 5:**
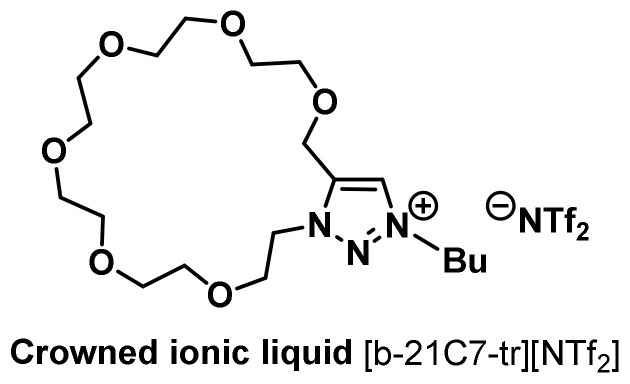
Structure of a crowned ionic liquid [b-21C7-tr][NTf_2_] [43].

Huang and coworkers introduced the structure of AIL with 1,4,7-triazacyclononane as the key affinity element, used for liquid–liquid extraction and purification of hexahistidine-tagged proteins [42]. This AIL could be readily regenerated using EDTA, followed by metal ion (Cu^+2^, Ni^+2^, and Zn^+2^) reincorporation. The M^+2^-AIL system showed high affinity toward the hexa(histidine)-tagged proteins so as to separate them from the protein mixture. In view of biological sample complexity, we reported the structure of crowned ILs (CILs) in 2016 [43]. We synthesized a series of novel types of CILs based on the intramolecular Huisgen 1,3-dipolar [3+2] cycloaddition reaction [44]. In addition to the known high affinity of crown ether with potassium ions, 18-crown-6 can be associated with protonated organoammonium cations to form stable complexes in both the gas and liquid phases (Figure 5). Some amino acids with a primary amine on the branch chain, such as lysine, can combine with 18-crown-6 to form a stable complex [45,46]. We introduced this concept into the CIL, mainly a functional IL with a bicyclic structure (Figure 5). The ring of triazolium provides this molecule with the special physical properties of IL, and crown ether ring sizes with different designs can control and influence the affinity with cations to achieve selective recognition and affinity bonding in the extraction of lysine- as well as arginine-rich peptides and proteins [43].

## 3. Ionic Liquids Can Be Thermally Responsive

### 3.1. Thermoresponsive Ionic Liquids (TILs) Are Smart Materials

Generally, conventional ionic compounds have good miscibility with water. In contrast, ILs with numerous combinations of anions and cations can have characteristics such as miscibility or separation with water due to the structural change in functional groups. Therefore, the differences in hydrophilicity and hydrophobicity are the most common method to classify the ILs: ionic liquids that are miscible with aqueous solutions are called hydrophilic ILs, and ionic liquids that are immiscible with water are called hydrophobic ILs. Recently, ILs as smart materials were discovered and developed, and the water solubility of these Ils, or ionic salts, dramatically changes upon temperature changes. These are named thermoresponsive ionic liquids (TILs). TIL indicates that the IL and any solvent are immiscible under a certain molar or mass ratio but can be turned into a single, homogeneous phase solution by raising or lowering the temperatures to reach their critical transition temperature (*T*_c_).

**Figure 6:**
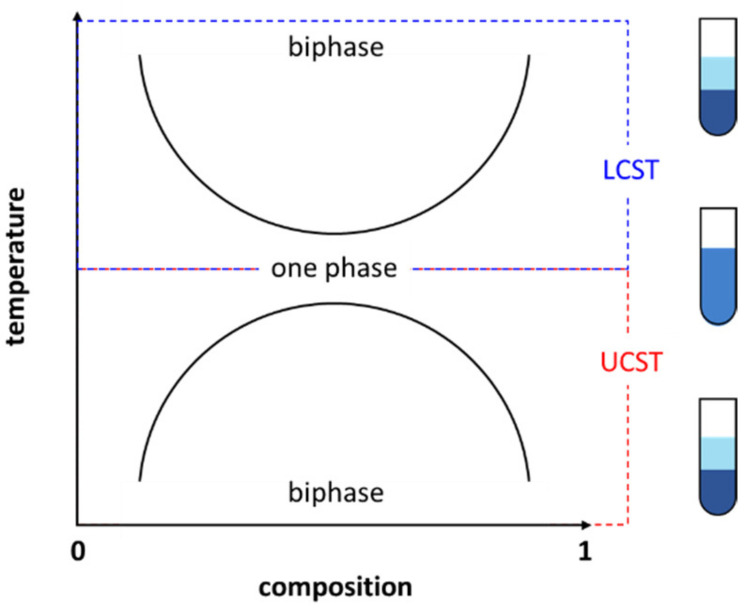
Schematic representation of a phase diagram showing the UCST (red) and LCST (blue) for thermoresponsive ionic liquid materials.

Generally, TILs with phase change behavior can be subdivided into two types [47]. Type I TILs reach their *T*_c_ with the increase in temperature. Then, all such types of substances are in a two-phase state above this temperature (that is, the minimum temperature at which they begin to be separated into two phases, called the lower critical solution temperature (LCST)) (Figure 6). Type II TILs reach their transition temperature with an increase in temperature. Then, all such types are in a mutual solubility state above this temperature (that is, the minimum temperature at which they begin to merge into one phase, called the upper critical solution temperature (UCST)) (Figure 6). Such solubility changes greatly provide application value, including seawater desalination [48], protein extraction and concentration [49,50,51,52], catalysis [53], and thermally regenerative electrochemical cycles [54]. Our review aims to focus on small-molecule TILs, and polymeric TILs are not discussed or included in this review.

### 3.2. Ionic Liquids Are Thermally Sensitive Materials

Hermann [55] first reported the thermoresponsive IL in 1989. He discussed the phase separation state of an aqueous electrolyte solution in batteries. He studied the equilibrium properties and thermodynamic changes of ILs qualitatively, during which it was observed that tetra-*n*-butyl ammonium thiocyanate [N_4,4,4,4_][SCN] has thermal sensitivity. By 1998, Dupont [56] reported the relationship between temperature and the solubility of ILs. This is the first literature on ILs with the UCST phenomenon. From then to 2001 and 2006, there were also relevant reports [57,58]. After 2007, Ohno [59,60] discovered the phosphonium IL-carrying LCST phase transition properties. In 2011, Ohno [61] developed [P_4,4,4,4_][Tf-Leu] for extracting proteins and biopolymers from water. In the subsequent year, Ohno began systematically studying the relationship between thermal sensitivity and IL composition. It was concluded from the experimental results that when the hydrophilicity was adjusted within a specific range by using phosphonium and ammonium cations combined with various anions, ILs may exhibit LCST phase behavior [62]. The work was also successfully applied to the extraction of cytochrome *c*.

**Figure 7:**
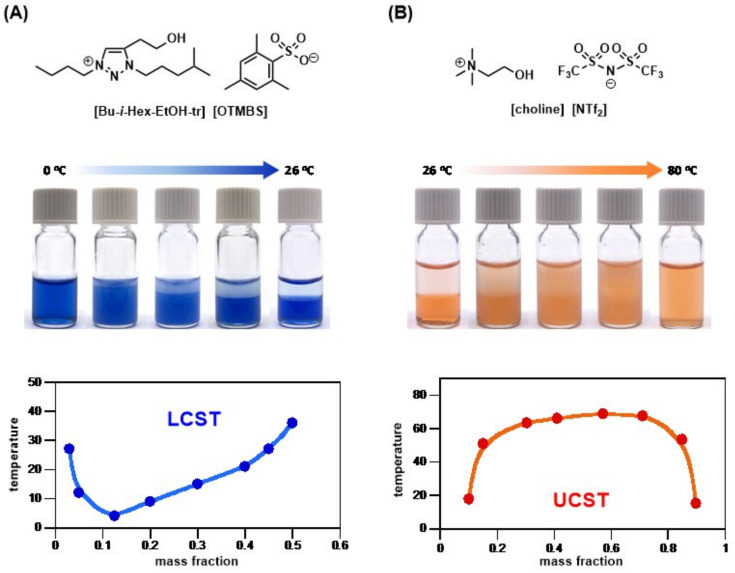
(**A**) Structure of a room-temperature ionic liquid [Bu-*i*-Hex-EtOH-tr][OTMBS] exhibiting LCST phase transition in water, and (**B**) the structure of the room-temperature ionic liquid [choline][NTf_2_] carrying UCST phase behavior with water (photos taken by Chien-Yuan Chen).

Since then, both UCST and LCST TILs have been extensively studied and discussed, attracting research from various fields to design and synthesize novel TILs. Nockemann [63] mixed [choline][NTf_2_] with water at a mass ratio of 1:1 and found that it exhibited UCST properties. [choline][NTf_2_] is immiscible with water at room temperature and becomes miscible when the temperature is raised above 72 °C. However, small-molecule ILs with LCST properties are much less common. This research on phosphonium ILs by Ohno was the most detailed study, which involved the investigation of various anions, such as sulfonate [62], fumarate and maleate [59], phosphonate [64], and carboxylate [65]. In addition to the seminal work conducted by Ohno, others began to search for new LCST TIL structures carrying both non-alkyl groups on cations and less common anions. Binnemans [66] introduced an amphoteric glyme motif in phosphonium cation and paired it with anions of *n*-alkyl and branched alkyl groups to facilitate the balance of overall hydrophobicity. The ILs reported display LCST phase transition in water, and, as expected, the *T*_c_ increases with the increase in length of the glyme structure. Wang [67] used [FeCl_4_]^−^ as the anion to prepare 16 magnetic ionic liquids, among which the cationic structures of ammonium, phosphonium, cholinium-liked, and imidazolium all showed the LCST thermoresponsive properties. They noted that cationic structure, alkyl chain length, and the molar ratio of FeCl_3_ to ionic liquid (the molar ratio of FeCl_3_/chloride ILs) significantly affected LCST phase behavior. For representative LUST and UCST small-molecule TIL examples from our laboratory, see Figure 7.

### 3.3. Zwitterionic Ionic Liquid Materials as TILs

**Figure 8:**
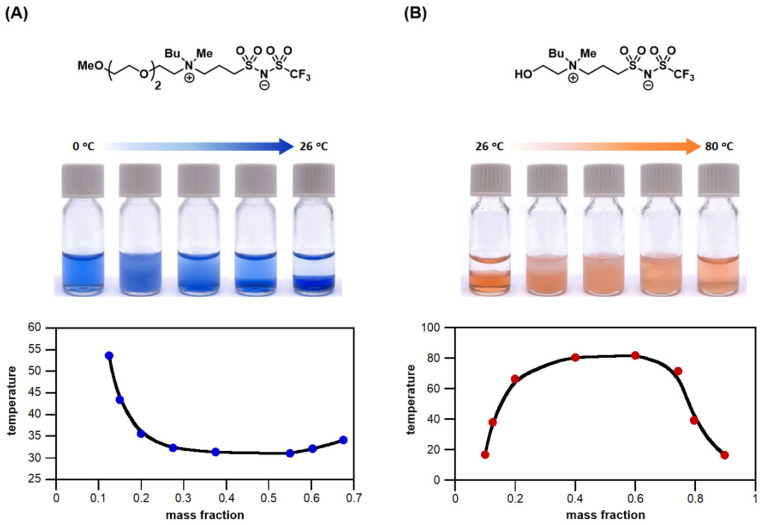
(**A**) Structure of a zwitterionic room-temperature ionic liquid [N_(mEG3)14_-C3-NTf] exhibiting LCST phase transition in water, and (**B**) the structure of a zwitterionic room-temperature ionic liquid [N_(EG1)14_-C3-NTf] carrying UCST phase behavior with water (photos taken by Chien-Yuan Chen).

In addition to the aforementioned thermoresponsive ILs, there were several zwitterionic ionic liquids (ZILs) reported in the literature that also displayed temperature sensitivity. In 2016, Ohno [68] published a ZIL from the reaction of trialkylamine with 1,3-propane sultone. When mixed with trifluoromethanesulfonic acid (HTfO), an LCST phase transition was observed. In 2018, Mochida [69] synthesized solvate ILs and protic ILs by taking alkyl aza-crown ethers as ligands to coordinate with metal ions and other anions. Among them, solvate ILs coordinated with Na showed the LCST properties at a specific concentration, and *T*_c_ has a downward trend with the increase in concentration. These examples demonstrated that the concentration of Brønsted acid and metal ions helps regulate the values of *T*_c_ more flexibly. Furthermore, the LCST systems can be the ideal application platform for catalytic reactions; that is, when the reactions are heated, the catalysts, such as water-soluble metal catalysts or enzymes, likely transfer from the aqueous phase to the IL-rich phase containing the substrates. After the reactions, the catalysts are then transferred back to the aqueous phase upon being cooled, and accordingly, the desired product is left in the IL phase alone. This temperature-responsive property facilitates rapid recycling and reuse of the catalysts, which is of great value to the reactions that need quantitative preparation in the industry [53]. For representative small-molecule LUST and UCST zwitterionic TIL examples from our laboratory, see Figure 8.

### 3.4. Mechanism of Thermal Sensitivity

The solubility of compounds in solvents is influenced by many noncovalent interactions, such as electrostatic force, Coulombic force, van der Waals, hydrogen bonding, and hydrophobic interactions. The physical properties of ionic liquids are uniquely different from those of common organic compounds. The overall strength of interaction forces in ILs may be varied by temperature changes, and accordingly, the dissolution of ILs in solvents can be altered dramatically, resulting in thermoresponsiveness. In addition, hydrogen bonds in ionic liquids are defined as “doubly ionic hydrogen bonds” [53], which are formed between two charged substances (cations and anions). This suggests that the types and numbers of hydrogen bond donor and receptor sites are varied, and a good number of distinct and unique hydrogen bonds are formed so that the solubility with solvents can be adjusted more effectively.

Cations and anions in ILs influence and contribute individually to their overall solubility in solvents. Typically, the anion plays a major role in the IL’s solubility with solvents [7]. By taking [NTf_2_] and [OMs] anions as examples with the same cations, the former is usually a hydrophobic IL, while the latter is a hydrophilic IL. The [NTf_2_] anion has a lower electron density, which leads to weak Coulombic force and hydrogen bonding; that is, the [NTf_2_] anion has a weak interaction with water in an aqueous solution, resulting in poor solubility. The influence of cation structure on solubility mainly comes from the hydrophobic interactions brought by alkyl or aromatic rings in the structure, which strengthen the intermolecular interactions between ILs themselves and depart from highly polar water solvents. The subtlety of ILs lies in that their designability is superior to that of conventional molecular solvents, and the strength of the interaction forces involved can be readily regulated through the modification of functional groups and the combination of ion pairs. All in all, the observation of thermoresponsiveness from ILs occurs under the condition that the interaction forces between cations, anions, and solvent molecules reach a critical balance. The temperature change can obliterate or alter those noncovalent forces by rearranging cations, anions, and water molecules [70,71]. Therefore, there is no definite and quantitative explanation of the causes of thermal sensitivity properties presently, and the molecular interaction forces of ILs with different structures can be diverse.

The mechanism of LCST is opposite that of UCST. Wang [72] chose five amino acid ILs to carry out all-atom molecular dynamics simulations, showing that for mixed amino acid ILs in water ([P_6668_]_1_[Lys]_0.5_[Asp]_0.5_/H_2_O) systems exhibiting LCST phase separation property, the amino or carboxylic acid group of one anion has hydrogen bond interaction with the carboxylate group of another anion in mixed systems. The strength of this hydrogen bond will also be affected by the temperature change, and the strength of the hydrogen bond in the anion will be enhanced when the temperature is raised. This also means the electrostatic interactions between anions and water are reduced, leading to poor solubility, temperature rise, and stratification. Therefore, we can sum up the causes of the ionic liquids of UCST and LCST. The intermolecular interactions of the former are stable at low temperatures but become weak and are eliminated with an increase in temperature so that they dissolve with the solvent. The interactions between the ions and solvent of the latter are relatively stable at low temperatures; the intermolecular noncovalent interactions are increased; and the solubility is reduced after heating, resulting in phase separation.

### 3.5. Combinatorial Discovery of Thermoresponsive Ionic Liquids

Before precise and rational prediction of TIL structures by reliable and quantitative calculation means can be realized, combinatorial chemistry may currently be the most convenient and effective technology platform to discover TIL expeditiously. Ammonium, phosphonium, and choline-based TILs previously reported by Ohno and Nockemann provide a preliminary understanding of the relationship between properties and structures of TILs, setting the stage for combinatorial library screening for TILs. Leveraging our extensive experience in structural design and functional ionic liquids and their various applications, we have accordingly developed and offered a rapid and efficient combinatorial approach for the design, synthesis, and discovery of TILs. Our recent deep involvement in small-molecule TIL research makes their expeditious discovery possible. Combinatorial chemistry is a technology to synthesize many compounds with the same core structure simultaneously within a short time with limited reaction steps. With this concept, we uncover the TILs with LCST or UCST properties quickly.

**Figure 9:**
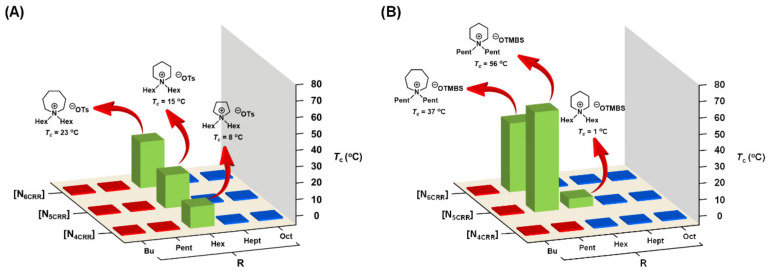
Phase transitions of a library of 30 ionic liquids upon mixing with water (1:2, *w*/*w*) at temperatures between 0 °C and 95 °C: (**A**) [OTs]-based ionic liquids and (**B**) [OTMBS]-based ionic liquids. Phase transition results shown in red and blue indicate an entirely homogeneous (one-phase) solution and heterogeneous (two-phase) mixture, respectively, between 0 °C and 95 °C [73].

**Figure 10:**
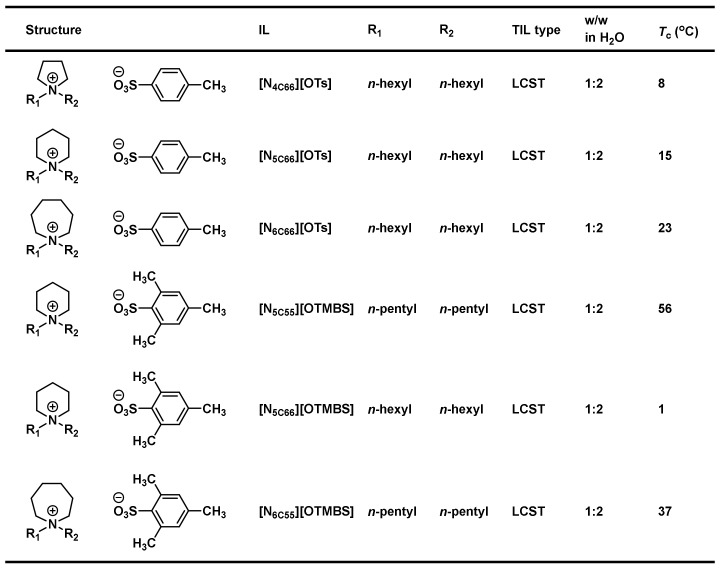
Six thermoresponsive ionic liquids were discovered from a library of 30 cycloammonium ILs.

In 2020, we first developed a library of 30 cycloammonium ILs carrying three heterocyclic amine cores (7-membered azepane, 6-membered piperidine, and 5-membered pyrrolidine) by combinatorial chemistry technology [73]. This IL library was purposely assembled through a convenient two-step synthesis so that it could be readily derivatized. After being mixed with water, six hydrated ILs were discovered from the library that exhibited phase transitions in water (Figure 9): [N_4C66_][OTs], [N_5C66_][OTs], [N_6C66_][OTs], [N_5C55_][OTMBS], [N_5C66_][OTMBS], and [N_6C55_][OTMBS] (*T*_c_ values were found between 1 °C and 56 °C), in which more immiscible two-phase mixtures were observed in cases of ILs with the hydrophobic [OTMBS] anion. It is worth mentioning that LCST TILs were indeed discovered to reside between completely homogeneous (single-phase) solutions and heterogeneous (two-phase) solutions. This result is consistent with the good balance between hydrophobicity and hydrophilicity previously disclosed by Ohno [62]. That is, TILs can be tailor-designed and structurally optimized according to the needs of their intended applications. We further confirmed that, with the mixing of a totally hydrophilic [N_4C55_][OTMBS] and a completely hydrophobic [N_4C66_][OTMBS] at a ratio of 1:1 (*w*/*w*), the thermoresponsive phase separation could be achieved through the hydrophilic–lipophilic interactions in water [73]. Lastly, as an unanticipated bonus, the overall thermoresponsiveness data of the IL library allowed a rapid assessment to suggest that [N_4C56_][OTMBS] (*T*_c_ = 19 °C) and [N_4C47_][OTMBS] (*T*_c_ = 37 °C) should carry, and indeed confirm, the presence of the thermal sensitivity phenomenon. In this work, we used cytochrome *c* (equine heart) and GFP (jellyfish aequorea victoria) for analysis to realize the power of TILs in enriching dilution protein concentrations [73]. Figure 10 summarizes our combinatorial discovery of 6 TILs from a small-molecule library of 30 cycloammonium ionic liquids.

**Figure 11:**
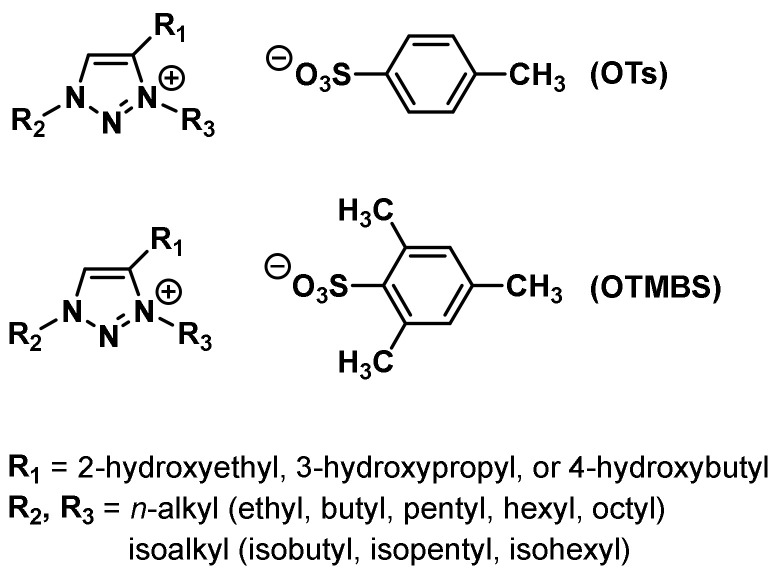
Structures of room-temperature 1,2,3-triazolium ionic liquids [74].

**Figure 12:**
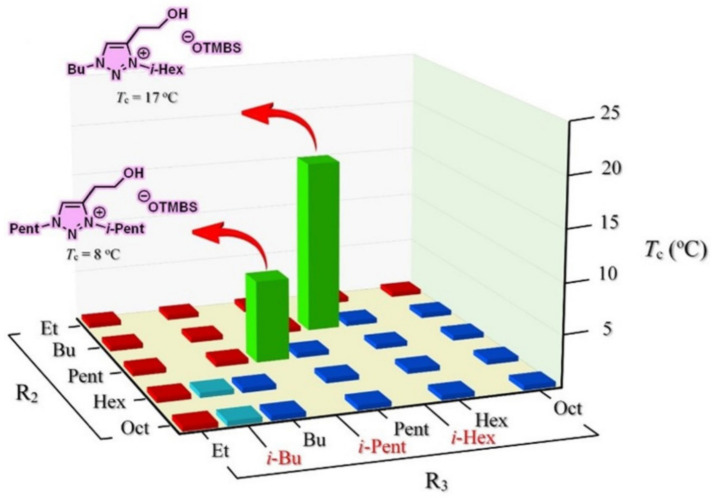
Thermoresponsiveness study of a library of 29 room-temperature 1,2,3-triazolium ionic liquids upon mixing with water (1:2, *w*/*w*) at temperatures between 2 °C and 90 °C. Phase transition results shown in red and blue indicate entirely homogeneous (one-phase) and heterogeneous (two-phase) solutions, respectively [74].

**Figure 13:**
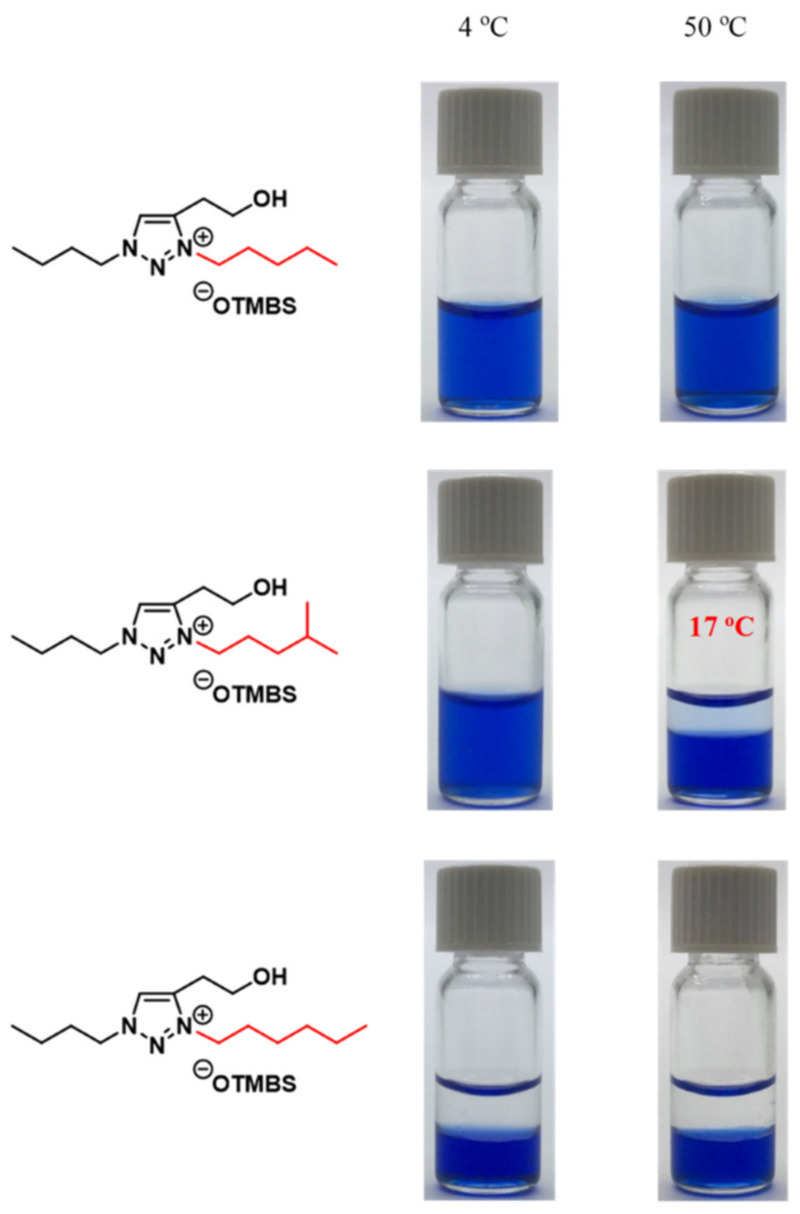
Temperature-dependent phase transitions of binary mixtures (2:1, *w*/*w*) of water with room-temperature ionic liquids [Bu-Pent-C2OH-tr][OTMBS], [Bu-*i*-Hex-C2OH-tr][OTMBS], and [Bu-Hex-C2OH-tr][OTMBS], respectively. The Coomassie brilliant blue R-250 (0.006 wt% in water) was added to accentuate the phase separation. Only [Bu-*i*-Hex-C2OH-tr][OTMBS] ionic liquid shows phase transition (*T*_c_ = 17 °C), whereas [Bu-Pent-C2OH-tr][OTMBS] and [Bu-Hex-C2OH-tr][OTMBS] are totally miscible and immiscible, respectively, with water at temperatures between 2 °C and 90 °C.

Albeit the majority of ionic liquid research has been on imidazolium-based ILs, we went ahead to investigate and research the thermoresponsive properties of 1,2,3-triazolium ILs, whose key triazole element can be readily assembled by click reaction. We accordingly synthesized a library of 160 ILs through our combinatorial approach (Figure 11) and ultimately discovered 22 LCST-type TILs [74]. At the beginning of our work, we synthesized a library of 50 [OTs]- and [OTMBS]-based 1,2,3-triazolium ILs (R_1_ = 2-hydroxyethyl, R_2_, R_3_ = ethyl, *n*-butyl, *n*-pentyl, *n*-hexyl, and *n*-octyl). Regrettably, none of the ILs carrying phase transition properties were found experimentally. After careful evaluation of the structure and phase separation (SPS) relationship between hydrophilic (one phase) and hydrophobic (two phases) ILs, we argued that if the R_3_ group was tuned further and altered a bit in its structure by replacing *n*-alkyl with a slightly more hydrophilic *iso*-alkyl group for ionic liquids that are on the rim between being totally hydrophilic and being totally hydrophobic, the phase transition should then be experimentally obtained. As expected, we were pleased that, with a slight polarity adjustment, two phase-transition ILs with LCST, namely [Bu-*i*-Hex-C2OH-tr][OTMBS] and [Pent-*i*-Pent-C2OH-tr][OTMBS] (Figure 12), were obtained (green label), with their *T*_c_ of 17 °C and 8 °C, respectively [74]. This successful structure engineering affirmed the tunability value of ionic liquids, demonstrating the potential of SPS (Figure 13). At the same time, we mixed a hydrophilic monophasic [Bu-Hex-C2OH-tr][OTs] with a hydrophobic biphasic [Bu-Oct-C2OH-tr][OTs] at a mass ratio of 1:1 and confirmed the thermal sensitivity phenomenon (*T*_c_ = 25 °C) [74]. This observation correlated well with the previous research results that the thermoresponsive property can be fine-tuned by regulating and optimizing the overall hydrophilicity and hydrophobicity of ILs. Overall, this combinatorial SPS platform allows a wide range of ionic salt combinations to quickly generate new TIL pairs without extensive structural fine-tuning and optimization [73,74].

**Figure 14:**
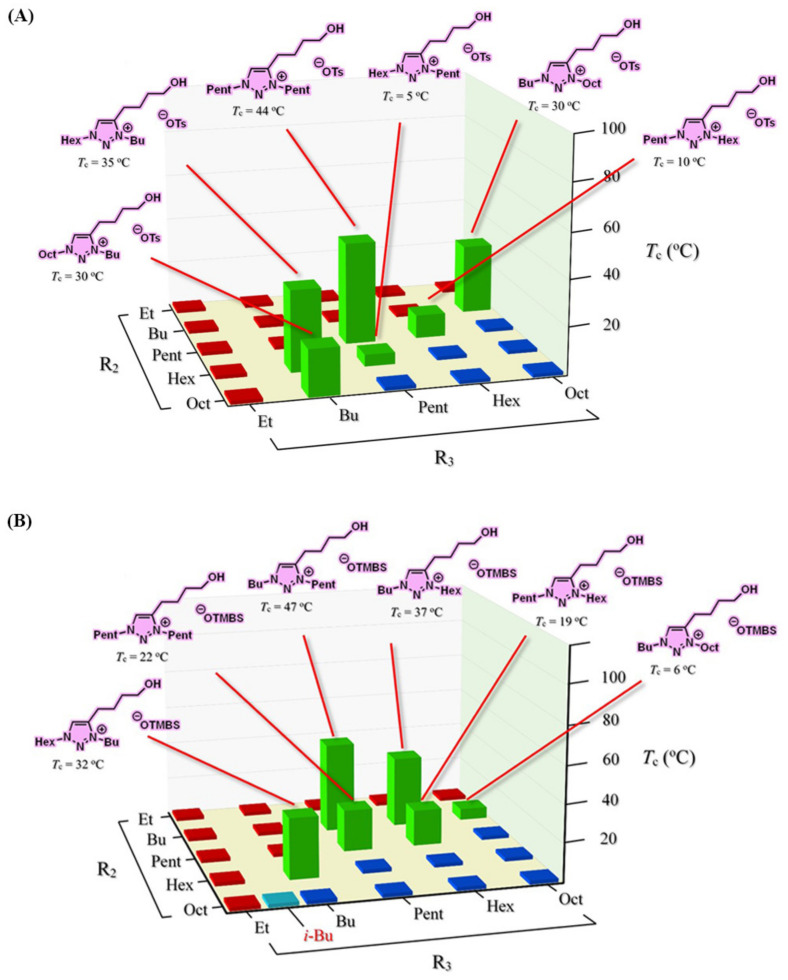
LCST Phase transitions of a library of 51 room-temperature 1,2,3-triazolium ionic liquids upon mixing with water (1:2, *w*/*w*) at temperatures between 2 °C and 90 °C: (**A**) OTs-based ionic liquids and (**B**) OTMBS-based ionic liquids [74].

**Figure 15:**
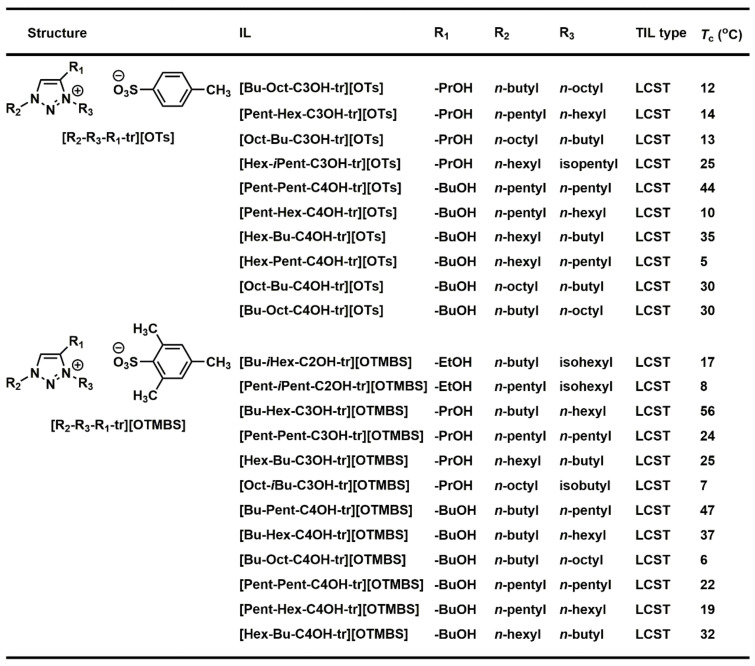
Twenty-two thermoresponsive ionic liquids were discovered from a library of 160 1,2,3-triazolium ILs.

We also successfully reported the high-yielding, 4-step synthesis of 52 ILs, [R_2_-R_3_-C3OH-tr][OTs] and [R_2_-R_3_-C3OH-tr][OTMBS], as the second library (Figure 10) [74]. In this library, the length of the R_1_ chain was extended to 3-hydroxypropyl. Eight ILs with LCST properties were experimentally discovered, among which two ILs carried a branched sidechain at R_3_: [Hex-*i*-Pent-C3OH-tr][OTs] and [Oct-*i*-Bu-C3OH-tr][OTMBS]. As expected, these eight ILs resided on the rim between being totally hydrophilic and being totally hydrophobic. *T*_c_ obtained were between 7 °C and 56 °C, showing their feasible applications in the biochemical field [74]. Finally, to further demonstrate the value and effectiveness of the combinatorial SPS platform developed, we expanded our study to a third library of 51 ILs, [R_2_-R_3_-C4OH-tr][OTs] and [R_2_-R_3_-C4OH-tr][OTMBS]. The total isolated yields of the library synthesis were high (44–71% and 42–69%, respectively) (Figure 14) [74]. In this 1,2,3-triazolium IL library, we extended the R_1_ sidechain further to 4-hydroxybutyl to fully control the hydration stability and the potential hydrogen bond capability. To our delight, 12 TILs (labeled in green) were discovered exhibiting LCST phase transition properties, among which 6 each are found in [OTs]- and [OTMBS]-based TILs (Figure 13). Identical to previous experimental results, these 12 ILs are in between a completely hydrophilic homogeneous phase (labeled in red) and a totally hydrophobic heterogeneous phase (labeled in blue). Moreover, these TILs discovered exhibit attractive low *T*_c_ (5 °C–47 °C), which should be desirable for low-energy applications. Figure 15 summarizes our combinatorial discovery of twenty-two TILs from three small-molecule libraries of 160 1,2,3-triazolium ionic liquids.

### 3.6. Combinatorial Discovery of Thermoresponsive Zwitterionic Ionic Liquids

**Figure 16:**
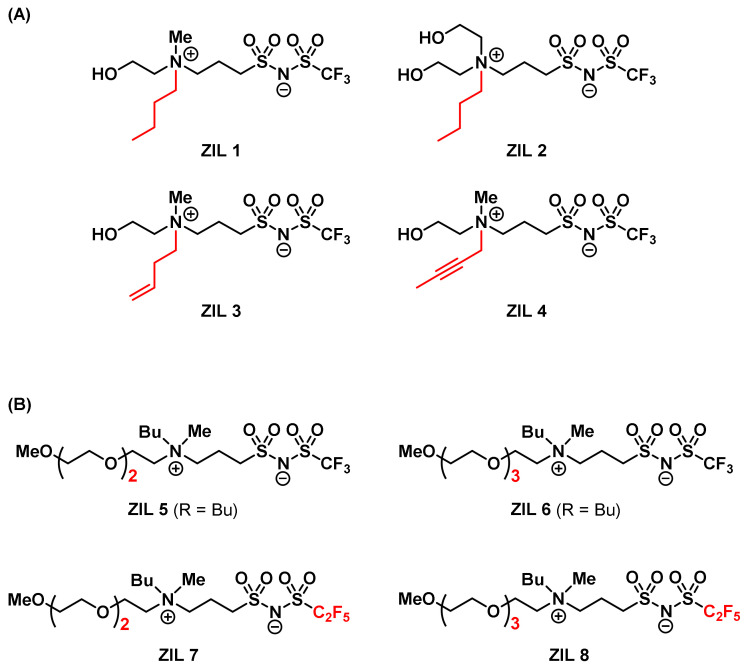
Structures of (**A**) UCST- and (**B**) LCST-type zwitterionic ammonium NTf- and NPf-based ionic liquids [52,75].

Inspired by Ohno’s work on ZILs in 2016 [68], we decided to explore combinatorially this type of molecular structure in the TIL field. We expected that the thermal sensitivity could be readily screened and obtained without any additional additives, such as Brønsted acids. As the first approach, we used [choline][NTf_2_] (*T*_c_ = 72 °C) previously reported from Nockemann Laboratory [63] as a starting point to design and structurally engineer ZIL materials by tethering together cation and anion covalently. The zwitterionic TIL materials should carry great potential as extraction solvents for biomolecules such as proteins since they differ from the IL problems often associated with ion exchanges during extraction. These structural characteristics of zwitterionic materials greatly reduce and may avoid the denaturation of proteins or the ion exchange of anions from ILs in the protein environment. Accordingly, we have synthesized and successfully developed two types of TILs having UCST as well as LCST phase transition properties by covalently linking ammonium cations with NTf anions (Figure 16) [52,75]. Moreover, their use for protein extraction was investigated.

**Figure 17:**
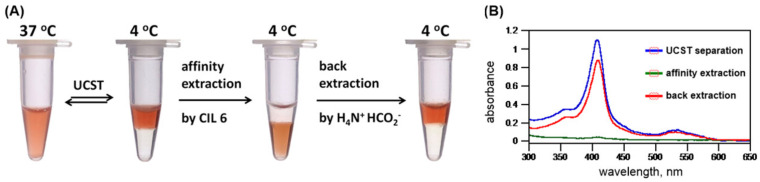
(**A**) Photos and (**B**) UV–Vis spectra of thermoresponsive phase separation were used to demonstrate affinity extraction of protein by a crowned ionic liquid [b-21C7-tr][NTf_2_] (163 mM) from its aqueous mixtures (1:1, *w*/*w*) of **ZIL 4** with a solution of equine heart cytochrome *c* (0.3 mM). After affinity extraction, competitive extraction of cytochrome c in the bottom IL layer into the upper aqueous phase could be readily achieved using ammonium formate (0.5 M). Efficiencies of extractions of cytochrome c were quantitatively measured in upper aqueous layers using the Soret band at 410 nm [52].

At the beginning of our UCST-TIL work, we constructed and synthesized a small library of twelve zwitterionic ionic liquids, among which only **ZIL 1** and **ZIL 2** exhibited UCST phase separation with water, and their *T*_c_ values were 84 °C and 10 °C (1:1, *w*/*w*), respectively. These *T*_c_ values were far from physiological temperature (37 °C) and would be unsuitable for applications in biomolecule extraction and biomolecular interaction analysis. Accordingly, further structural engineering and optimization were made on **ZIL 1** and **ZIL 2**. *T*_c_ values in UCST systems are decreased by increasing overall hydrophilicity in structure, and the polarity of substituents (e.g., polarity: alkyl < alkenyl < alkynyl group) on ZIL structure also influences the overall hydrophilicity without changing the number of carbon atoms. Therefore, we amended the saturated alky chain to the unsaturated, slightly more polar alkenyl and alkynyl groups (Figure 16A). To our delight, the newly synthesized **ZIL 3** and **ZIL 4** were found to exhibit lower *T*_c_ values (53 °C and 33 °C, respectively) [52], among which **ZIL 4** is well suited for the application of protein experiments, allowing for temperature-guided affinity extraction without altering protein stability. As shown in Figure 17, by taking cytochrome *c* as an example, affinity extraction through the temperature phase transition was readily achieved with a crowned ionic liquid (Figure 5) previously developed in our laboratory.

**Figure 18:**
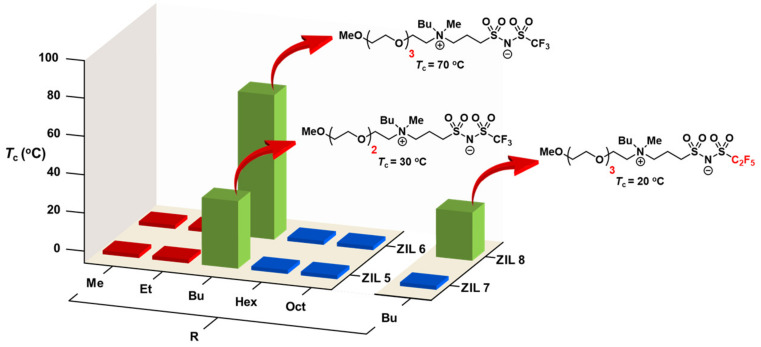
Upon being mixed with water (1:2, *w*/*w*) at temperatures between 4 °C and 90 °C, **ZIL 5**, **ZIL 6,** and **ZIL 8** showed LCST-phase transitions in water [75].

In addition, by replacing the hydroxyl group in the ZIL structure (Figure 16A) with a methoxy group (Figure 16B), we limited the hydrogen bond capability that was critical to the UCST thermoresponsive property and greatly reduced the hydrophilicity of ZILs. In order to compensate for and balance the lost hydrophilicity, we selected triglyme and tetraglyme groups to replace the ethanol side chain in the UCST ZIL structure. Then, we adjusted the hydrophobic ability by the length of the alkyl carbon chain (Figure 14B). In the library of ten ZILs, two ZILs were discovered to carry LCST phase separation in water: **ZIL 5** (*T*_c_ = 30 °C) and **ZIL 6** (*T*_c_ = 70 °C) (Figure 18).

**Figure 19:**
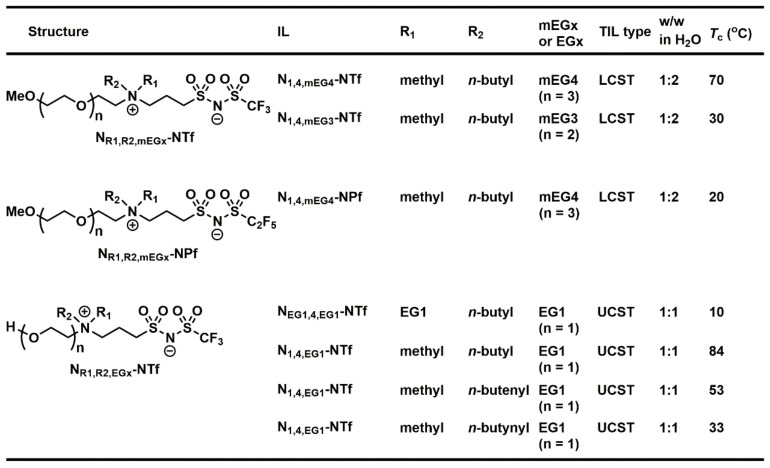
Seven TILs were discovered from two small-molecule libraries of twenty-eight ZILs [75].

In the afore-discussed UCST ZIL work [52], we optimized thermoresponsive properties by engineering the polarity of the cations (Figure 14A). In this LCST-ZIL work [75], we obtained favorable *T*_c_ values by fine-tuning the hydrophobicity of the anion (Figure 16B). In order to obtain biocompatible *T*_c_ values for LCST ZILs, the trifluoromethyl (NTf) group in both **ZIL 5** and **ZIL 6** was substituted with a more hydrophobic pentafluoroethyl (NPf) group in **ZIL 7** and **ZIL 8**. Ultimately, **ZIL 8** was successfully engineered and found to exhibit an attractive *T*_c_ (20 °C) for affinity extraction and biomolecular interaction analysis [75]. Figure 19 summarizes our combinatorial discovery of seven TILs from three small-molecule libraries of twenty-eight zwitterionic ammonium NTf- and NPf-based ionic liquids.

### 3.7. Future Prospect of Thermoresponsive Ionic Liquid Materials

Due primarily to their negligible volatility and recyclable characteristics, ionic liquids were at first considered or even favored by many scientists as green solvents. It was then quickly realized that important issues such as environmentally friendly and less toxic ILs should be investigated and addressed before they can be classified as green solvents. Despite the lack of air pollution from ILs, our recent close research collaboration with researchers from various fields has revealed the importance of cytotoxicity and biosafety studies of ILs [11]. When ILs are put into industrial production and a small amount of IL goes into the ecosystem, it should cause harm to the environment and directly endanger human health [76,77,78,79]. Therefore, the structural design of ILs in modern times should not only meet the experimental requirements but also consider environmental issues of toxicity and safety toward organisms, which is also one of the aims of IL research and development in our laboratory.

The physical and chemical properties of ZIL materials are uniquely different from those of common Ils in that ZILs are neutral in charge and known to exhibit very high polarity. Compared with ILs, whose studies are largely established, the research and development of small-molecule ZILs remain mostly unexplored. Albeit we successfully developed zwitterionic NTf-based TILs [52,75], this NTf anion nevertheless is hydrophobic in structure and likely in close association with cytotoxicity. Accordingly, it is necessary to structurally design and develop a more environmentally friendly, thermoresponsive ZIL. Since the core of biocompatible sulfobetaine is less cytotoxic [76,80], we reported in 2023 the synthesis and discovery of a small library of sixteen sulfobetaine zwitterionic materials that exhibit LCST phase transitions in water [51]. We engineered to fine-tune overall ZIL structure hydrophobicity by introducing benzyl, mesityl, and 4-bromobenzyl substituents as sidechain groups to construct fifteen ZILs, of which seven ZILs were completely miscible in water, six ZILs formed two phases in water, and two ZILs were found to exhibit LCST properties with high *T*_c_ values (84 and 60 °C). To obtain an attractive *T*_c_ value for immediate biomolecular extraction and purification, it is necessary to further increase its hydrophobicity in the ZIL structure. Consequently, the side chain group in the ZIL structure was optimized to a 3,5-dibromobenzyl group (*T*_c_ = 38 °C). This optimization eventually led to the successful enrichment of an organic dye and human hemoglobin by 16 and 11 folds, respectively.

## 4. Conclusions

This review primarily focused on the recent development of ionic liquids. It put forward many applications, including supported, task-specific, functionalized, affinity, and thermoresponsive ionic liquids (TILs). In this review, we introduce the latest development of TIL in detail. This covers their unique temperature-switchable properties and principles, mechanisms of action, major differences between thermoresponsive ILs and ZILs, and finally, our most recent combinatorial library, screening, and discovery of thermoresponsive IL-based materials as new smart materials. These small-molecule TILs typically exhibit LCST or UCST phase transitions in water. They could also rationally fine-tune, engineer, and eventually optimize their structures to afford TILs displaying desired *T*_c_ values attractive for potential applications.

**Figure 20:**
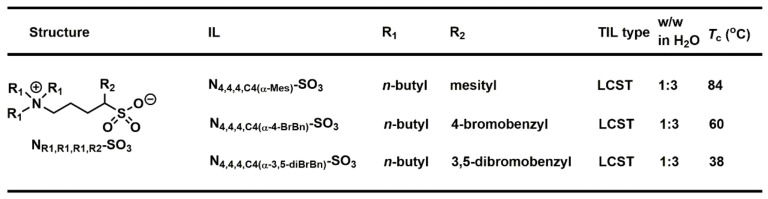
Three thermoresponsive materials were discovered from a small-molecule library of sixteen zwitterionic ammonium sulfonates.

We concur that before precise machine learning prediction of TIL structures can be realized, combinatorial chemistry may currently be the most convenient and effective technology platform to discover TIL expeditiously. Through our rational TIL design and combinatorial library synthesis and screening, we have successfully discovered novel chemical structures of TILs (Figure 10, Figure 15, Figure 19 and Figure 20). We have also fine-tuned their structures and *T*_c_ temperatures for proof-of-concept applications in biomolecular interaction analysis and affinity extraction, which will have great prospects for future biological applications. Without a doubt, we shall continue developing new TIL structures and studying their applications related to ILs and TILs.
